# Altered knee kinematics after posterior cruciate ligament single-bundle reconstruction—a comprehensive prospective biomechanical *in vivo* analysis

**DOI:** 10.3389/fbioe.2024.1322136

**Published:** 2024-01-25

**Authors:** Stephan Oehme, Philippe Moewis, Heide Boeth, Benjamin Bartek, Christoph von Tycowicz, Rainald Ehrig, Georg N. Duda, Tobias Jung

**Affiliations:** ^1^ Center for Musculoskeletal Surgery, Charité–Universitätsmedizin Berlin, Berlin, Germany; ^2^ Berlin Institute of Health at Charité–Universitätsmedizin Berlin, Julius Wolff Institute Berlin, Berlin, Germany; ^3^ Zuse Institute Berlin, Berlin, Germany

**Keywords:** prospective case series PCL, posterior cruciate ligament, posterior cruciate ligament reconstruction, gait analysis, knee biomechanics

## Abstract

**Purpose:** Passive tibiofemoral anterior-posterior (AP) laxity has been extensively investigated after posterior cruciate ligament (PCL) single-bundle reconstruction. However, the PCL also plays an important role in providing rotational stability in the knee. Little is known in relation to the effects of PCL single-bundle reconstruction on passive tibiofemoral rotational laxity. Gait biomechanics after PCL reconstruction are even less understood. The aim of this study was a comprehensive prospective biomechanical *in vivo* analysis of the effect of PCL single-bundle reconstruction on passive tibiofemoral rotational laxity, passive anterior-posterior laxity, and gait pattern.

**Methods:** Eight patients undergoing PCL single-bundle reconstruction (seven male, one female, mean age 35.6 ± 6.6 years, BMI 28.0 ± 3.6 kg/m^2^) were analyzed preoperatively and 6 months postoperatively. Three of the eight patients received additional posterolateral corner (PLC) reconstruction. Conventional stress radiography was used to evaluate passive translational tibiofemoral laxity. A previously established rotometer device with a C-arm fluoroscope was used to assess passive tibiofemoral rotational laxity. Functional gait analysis was used to examine knee kinematics during level walking.

**Results:** The mean side-to-side difference (SSD) in passive posterior translation was significantly reduced postoperatively (12.1 ± 4.4 mm vs. 4.3 ± 1.8 mm; *p* < 0.01). A significant reduction in passive tibiofemoral rotational laxity at 90° knee flexion was observed postoperatively (27.8° ± 7.0° vs. 19.9° ± 7.5°; *p* = 0.02). The range of AP tibiofemoral motion during level walking was significantly reduced in the reconstructed knees when compared to the contralateral knees at 6-month follow-up (16.6 ± 2.4 mm vs. 13.5 ± 1.6 mm; *p* < 0.01).

**Conclusion:** PCL single-bundle reconstruction with optional PLC reconstruction reduces increased passive tibiofemoral translational and rotational laxity in PCL insufficient knees. However, increased passive tibiofemoral translational laxity could not be fully restored and patients showed altered knee kinematics with a significantly reduced range of tibiofemoral AP translation during level walking at 6-month follow-up. The findings of this study indicate a remaining lack of restoration of biomechanics after PCL single-bundle reconstruction in the active and passive state, which could be a possible cause for joint degeneration after PCL single-bundle reconstruction.

## Introduction

The posterior cruciate ligament (PCL) is the primary stabilizer against posterior tibial loads of the knee joint (Sekiya et al., 2008). It acts as a secondary stabilizer against rotational loads, especially at 90° knee flexion (Winkler et al., 2021a). PCL tears are a rare injury with an estimated annual incidence of 1.8 per 100,000 capita (Sanders et al., 2017). Approximately 60% of PCL injuries are associated with additional ligamentous lesions (Owesen et al., 2017). The most common associated injuries are posterolateral corner (PLC) injuries, which occur in 38%–73% of PCL injuries (Anderson et al., 2018). PCL tears are devastating injuries that lead to chronic instability of the tibiofemoral joint and if left untreated lead to degeneration of the medial tibiofemoral and patellofemoral compartment (Strobel et al., 2003). PCL single-bundle reconstruction is an established procedure to restore knee joint kinematics. It strives to restore increased tibiofemoral anterior-posterior (AP) laxity and leads to satisfying functional outcomes (Schroven et al., 2022). The effects of PCL reconstruction on tibiofemoral AP-laxity have been thoroughly investigated (Winkler et al., 2021b). Measurement of AP-laxity via stress radiography is used to ascertain the severity of a PCL insufficiency and the success of PCL reconstruction in clinical practice (Jung et al., 2006a). Passive tibiofemoral axial rotational laxity after PCL reconstruction or combined PCL and PLC reconstruction is also analyzed in several biomechanical cadaveric studies (Harner et al., 2000; Krudwig et al., 2002; Sekiya et al., 2005; Markolf et al., 2007; Apsingi et al., 2008a; Wijdicks et al., 2013; Kennedy et al., 2014). Very few *in vivo* studies examined passive tibiofemoral axial rotational laxity after PCL reconstruction, and this was only performed with additional PLC reconstruction (Fanelli and Edson, 2004; Kim et al., 2013; Zorzi et al., 2013). The effect of PCL reconstruction on gait biomechanics is even less understood (Tibone et al., 1988; Hart et al., 2009; Brisson et al., 2021). The aim of the present study is to gain a comprehensive understanding of *in vivo* knee kinematics after PCL single-bundle reconstruction. We hypothesized that PCL single-bundle reconstruction with optional PLC reconstruction restores increased passive tibiofemoral AP laxity and increased passive tibiofemoral rotational laxity in patients with PCL insufficiency. Furthermore, we hypothesized that the PCL reconstructed knees do not show significantly altered knee kinematics during level walking compared to the contralateral knees. Altogether this study aims to provide, for the first time, a comprehensive prospective *in vivo* analysis of knee biomechanics in the passive and active state in patients undergoing PCL single-bundle reconstruction.

## Materials and methods

### Patient cohort

**FIGURE 1:**
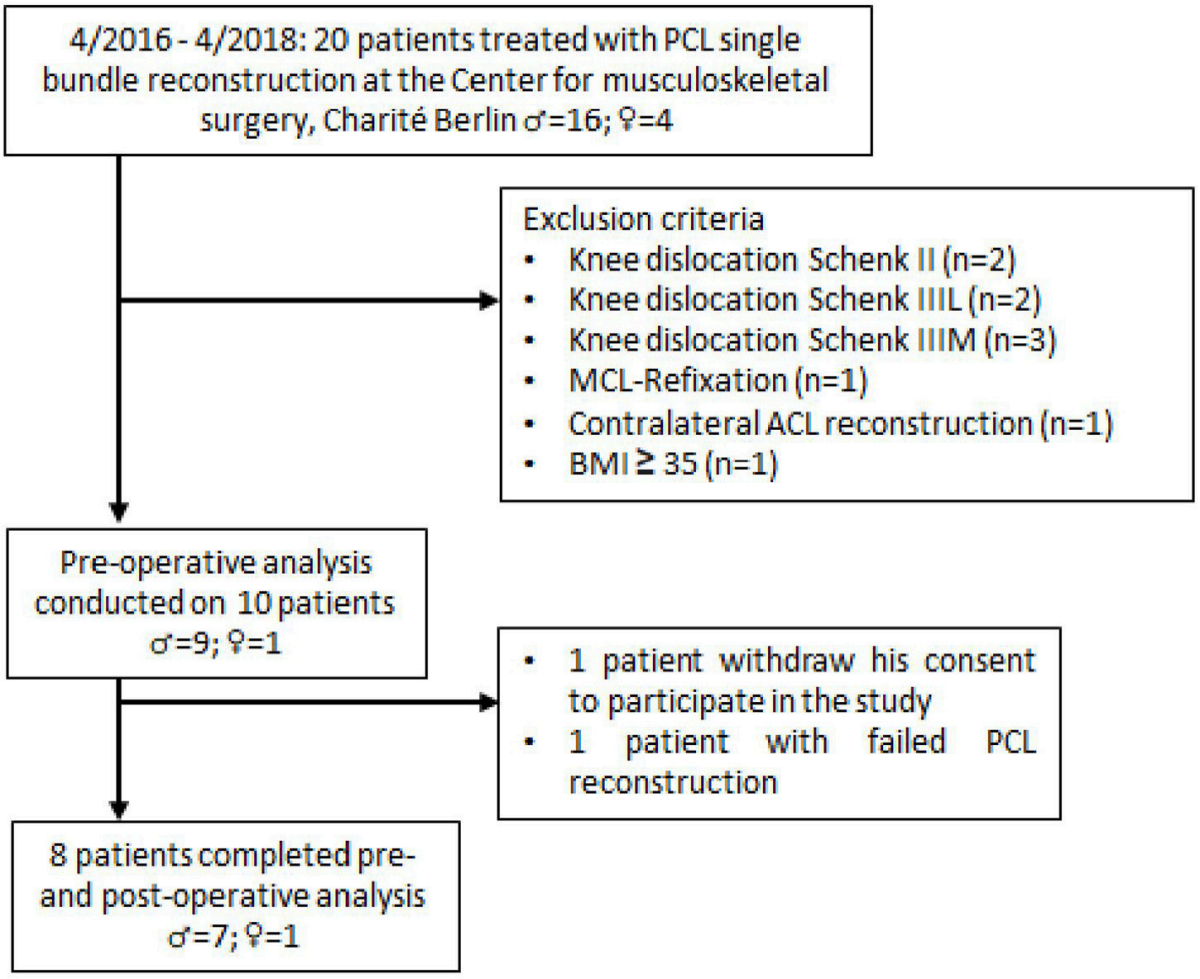
CONSORT diagram of patient acquisition.

Twenty patients were initially identified for potential enrollment in this prospective, single-center study. Eight patients met the inclusion criteria and were enrolled in the study (Figure 1). PCL insufficiency was determined using stress radiography. A partial PCL tear was defined as < 8 mm of increased posterior tibial translation, isolated complete PCL tears as 8–12 mm increased posterior tibial translation, and combined complete PCL tears as > 12 mm increased posterior tibial translation compared to the contralateral (CL) healthy knee (LaPrade et al., 2015). Indication for additional PLC reconstruction was an increased posterior tibial translation >12 mm compared to the contralateral knee or a difference of >10° between knees on the dial test at 30° and/or 90° knee flexion and moderate or severe instability in varus stress compared with the uninvolved knee (Figueroa et al., 2021).

All patients had a chronic PCL insufficiency with a history of a PCL injury of more than 3 months ago. Exclusion criteria included previous PCL injury, PCL avulsion fracture, body mass index (BMI) ≥ 35 kg/m^2^, concomitant ligament insufficiency (excluding PLC insufficiency), knee dislocation, previous knee surgery, history of ligament injury of the contralateral knee, flexion-extension limitations in the contralateral knee, osteoarthritis ≥ grade II by Kellgren and Lawrence, and pregnancy. The average age of the patients was 35.6; ±6.6 years, and the mean BMI was 28.0; ±3.6 kg/m^2^. Measurements took place preoperatively and 6 months postoperatively (∅ 5.2 ± 0.7 months). All patients underwent preoperative clinical examination and operative treatment with a unilateral primary PCL single-bundle reconstruction by a single surgeon (TJ). PCL single-bundle reconstruction was performed with ipsilateral five-strand semitendinosus and gracilis autograft in seven patients. One patient received an Achilles tendon allograft. The conventional Arthroscopically-assisted tibial tunnel technique was used in all patients (Jung et al., 2006b). Additionally, PLC reconstruction in modified Larson technique with contralateral gracilis autograft was performed in three patients. A standard rehabilitation protocol was applied to all patients. It consisted of knee immobilization in a straight posterior tibial support splint for 6 weeks, following an application of a PCL dynamic brace with gradual enabling of the range of motion for the next 6 weeks. Mobilization under 15 kg partial weight bearing on forearm crutches was performed during the first 6 weeks with subsequent transition to full weight bearing. Passive flexion exercises were started postoperatively, which were carefully increased to 90° knee flexion by the end of the sixth week. Active and passive mobilization beyond 90° of flexion was permitted after the sixth week. The study was approved by the local ethics committee (Ethikkommission der Charité - Universitätsmedizin Berlin; Nr: EA2/141/14). All subjects provided written informed consent prior to participation and were properly informed about the different measurement procedures.

### Measurement of passive axial rotational knee joint laxity

**FIGURE 2:**
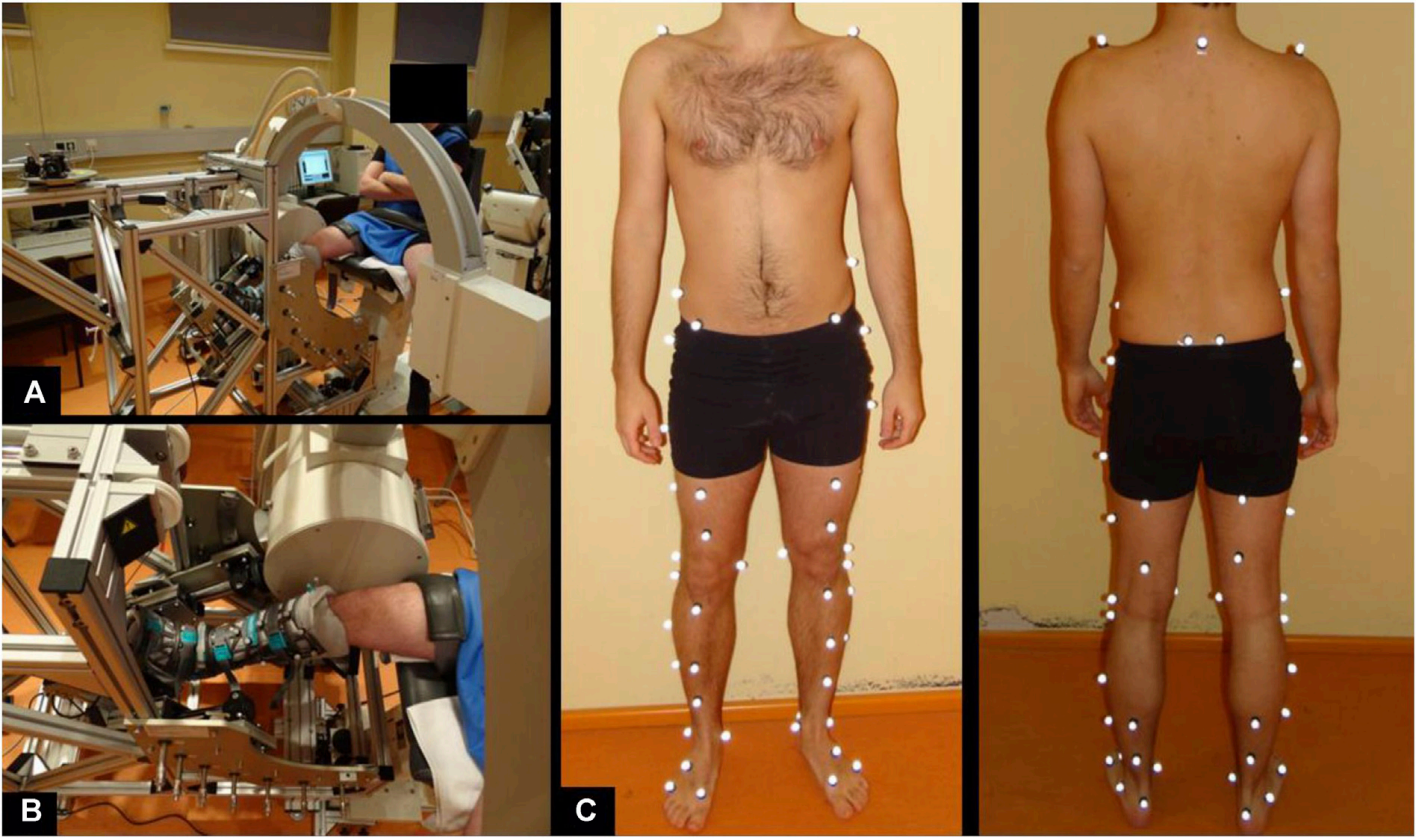
Measurement setup to assess passive knee joint rotation **(A, B)** and marker set of motion gait analysis to determine in-gait knee joint rotation and translation **(C)**.

A certified rotometer device (REF; Berlin CERT, certification number: Z-11-131-MP) was used for the accurate and objective measurement of passive rotational laxity. The device allows a progressive and controlled application of axial internal and external rotation of the tibiofemoral joint at a knee flexion angle range of 0°–90°. Details on technical specifications as well as measurement procedure have been described previously (Moewis et al., 2014; Moewis et al., 2016). A maximum internal and external torque of 3 Nm was applied to conduct a gentle testing of passive tibiofemoral rotational stability applied in the clinical dial test. Moreover, the applied torque was also chosen since it has been used in several *in-vitro* studies (Kaneda et al., 1997; Wiley et al., 2006). A C-arm X-ray fluoroscopic device was used to record the axial rotational movement during synchronized application of the external axial torque (Figure 2A, B). The X-ray images were collected at 3 Hz during a complete axial rotation cycle. The distortion of the images was corrected with a previous calibration procedure (Garling et al., 2005). The use of X-rays was approved by the German Federal Office for Radiation Protection (Bundesamt für Strahlenschutz, Approval Number: Z5-22462/2-2014–096). Measurements were conducted at 90° of knee joint flexion considering the higher contribution of the PCL towards rotational stabilization at higher flexion degrees (Sekiya et al., 2005; Charbonnier et al., 2020).

### Measurement of passive translational knee joint laxity

Conventional stress radiography (Telos Stress Typ GA-III/E, Telos GmbH, Wölfersheim-Berstadt) was used to collect stress images of both knee joints to quantify the passive posterior and anterior tibial displacement in a side-by-side comparison. The procedure was conducted at 90° of knee joint flexion. A defined force of 150 N was applied anteriorly and posteriorly to press the tibia into an anterior and posterior drawer respectively. Four X-ray images for posterior and anterior drawer of both legs were generated to determine the side-to-side difference (Jung et al., 2006a).

### Measurement of active knee joint kinematics

We chose to use level walking for the assessment of active knee joint kinematics as it is a common daily activity that can be conducted properly by patients with PCL insufficiency. The kinematics was assessed by means of 59 reflective markers (Taylor et al., 2010) (Figure 2C). The spatial position of these markers was tracked at 120 Hz using an infrared optical motion capture system (10 T20S cameras, Vicon, Oxford, United Kingdom). The subjects were asked to perform 10 repetitions along a 10 m pathway at a self-selected speed.

### Fluoroscopic analysis and quantification of skeletal passive tibiofemoral rotation

An analysis-by-synthesis approach was adapted to calculate the motion of the skeletal joint structures recorded during the fluoroscopic analysis. The first step of this approach consisted of a pseudo-computed tomography (Pseudo-CT) synthesis. During this step, the subject-specific 3D anatomy of the bony anatomy of the knee was determined via magnetic resonance imaging (MRI). The MRI specifications were the following: Proton density-weighted MRI scans, slice thickness 0.6 mm; voxel size 0.46 mm × 0.46 mm x 0.6 mm; time to repeat (TR) 1,200 ms, time to echo (TE) 36 ms; flip angle 120°; 160 slices. To extract the bones’ shape, a convolutional neural network with U-net architecture in combination with statistical shape models (SSM) as anatomical prior for regularization was employed (Ambellan et al., 2019). To augment the subject-specific shape model with electron density information an atlas-based approach in terms of a pre-trained statistical shape and intensity model (SSIM) was used (von Tycowicz et al., 2018). Image-based 3D/2D (three-dimensional/two-dimensional) registration (Markelj et al., 2012) was applied to determine the transformation, i.e., position and orientation of the derived bone shapes within the fluoroscopic images. This procedure provides sub-millimeter and sub-degree accuracies for in-plane translation and rotation (Penney et al., 1998). This methodology builds on a previous approach with the following accuracy: translation: 0.4–0.9 mm in-plane, 2.6–9.3 mm out-of-plane; rotation: 0.5°–1.9° (Moewis et al., 2012). Manual positioning was conducted for the first frame of each fluoroscopic sequence followed by extrapolation to propagate the position to the subsequent frames.

Torque-rotation curves were generated for each pre- and postoperative measurement, the applied external axial torque, and the calculated axial rotation from the fluoroscopic analysis. The peak rotations at 3 Nm were used as a measure of internal and external rotational laxity. To correct for the effect of each subject’s natural knee rotation angle, the neutral reference rotation for each subject was determined as the average angle at which zero resistance to rotation was observed (taking both the internal and external rotations into consideration). These neutral reference positions were then used for group-wise analyses (Oehme et al., 2022).

### Stress radiography analysis

The Jacobsen technique (Jacobsen, 1976) was used to quantify the posterior and anterior drawer of the PCL insufficient, reconstructed, and contralateral knees during the stress radiography. Peripheral bony landmarks are used to determine the tibial displacement relative to the femur. First, a straight line is drawn along the medial tibial plateau. Then, two perpendicular lines are drawn onto the tibial plateau, starting from the center of the most posterior medial and lateral contours of the femoral condyle and the center of the most posterior edges of the medial and lateral tibial plateau. The distance between these perpendiculars represents the total posterior displacement, which is measured in millimeters (Jung et al., 2006a). The measurements were performed independently by two examiners [BB, SO]. The mean of both results was then determined. Failed PCL reconstruction was defined as a reduction of less than 50% of the SSD between the insufficient knee and the contralateral knee.

### Analysis of tibiofemoral rotation and translation during walking

The optimal common shape technique (OCST), the symmetrical axis of rotation approach (SARA) and the symmetrical center of rotation estimation (SCoRE) were applied to quantify the relative tibiofemoral rotation and translation (Taylor et al., 2010; Ehrig et al., 2011). The calculated 3D tibiofemoral motion data during self-paced walking was split into multiple repetitions of individual gait cycles (Sharenkov et al., 2014). For each kinematic variable, 101 discrete points according to 0%–100% (heel strike to heel strike) of the gait cycle were extracted at 1% intervals using interpolation. The range of motion (RoM) for the AP translation and axial rotation was calculated as the difference between the minimum and maximum AP translational and rotational movement of the thigh and shank segments coordinate systems relative to one another. The kinematic curves were averaged across trials for each patient as well as across all patients in each cohort to determine group differences between PCL insufficient knees, contralateral knees and PCL reconstructed knees.

### Statistical analysis

An *a priori* power analysis was performed for the passive rotational knee joint measurements based on a previous study using the same rotometer device for the analysis of passive rotational knee joint laxity (Moewis et al., 2016). The analysis resulted in a calculated sample size of five subjects to achieve a statistical power of 1-beta = 0.80 and an alpha of 0.05. The data were tested for normal distribution by Shapiro-Wilk-Test. The normal distribution was confirmed. For group comparison, paired t-tests for peak values and ranges was conducted when comparing the injured and reconstructed knees and unpaired t-test when comparing to the contralateral healthy knees. The respective t-tests were also conducted on the gait analysis data, specifically for the heel strike event as well as the maximum and minimum values. All statistical analyses were accomplished using IBM SPSS Statistics 25 (Chicago, Illinois, USA).

## Results

The results are collected in Table 1.

**TABLE 1:** Peek and range values fro the contralateral, PCL-insufficient and PCL-reconstructed knees. Bold *p* values indicate the comparison to the contralateral knees.

	Contralateral (mean ± sd)	PCL-insufficient (mean ± sd)		PCL-reconstructed (mean ± sd)	
Passive rotational knee joint laxity (°)	21.8 ± 4.6	27.8 ± 7.0	p = 0.03	19.9 ± 7.5	**p = 0.28**
					p = 0.01
Passive translational knee joint laxity (mm)	4.8 ± 1.0	16.9 ± 4.0	p < 0.01	8.9 ± 2.2	**p < 0.01**
					p < 0.01
Tibiofemoral rotational motion (°)	15.2 ± 3.8	16.1 ± 3.9	p = 0.31	15.3 ± 3.2	**p = 0.47**
					p = 0.29
Tibiofemoral anterior-posterior motion (mm)	16.6 ± 2.4	17.0 ± 5.8	p = 0.44	13.5 ± 1.6	**p < 0.01**
					p = 0.07

### Passive rotational knee joint laxity

**FIGURE 3:**
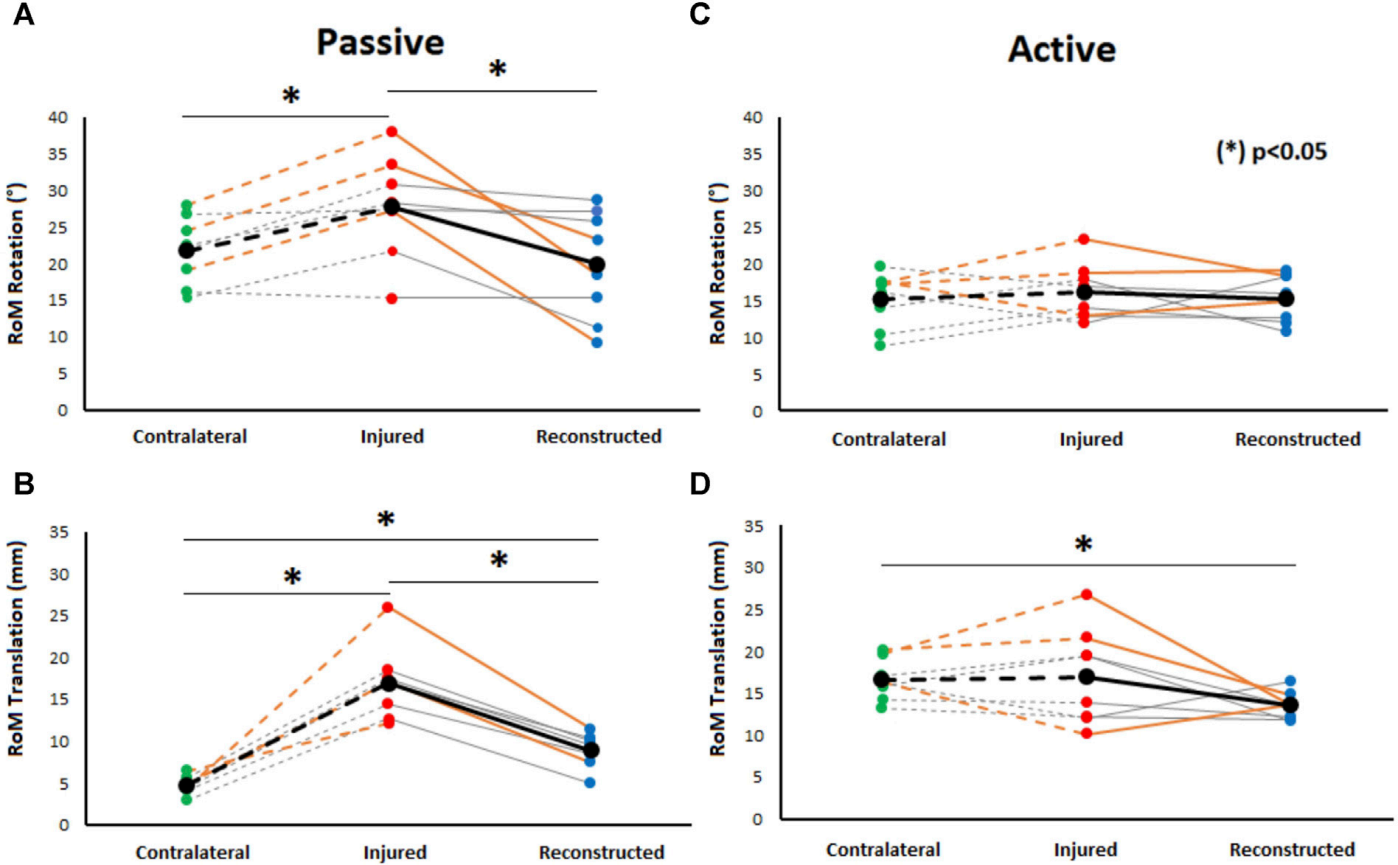
Comparison of passive rotational (at 90° knee flexion) **(A)** and passive translational tibiofemoral RoM **(B)** to active rotational **(C)** and translational tibiofemoral RoM **(D)** during gait between healthy contralateral, PCL insufficient, and reconstructed knees. Significant differences between PCL insufficient knees, CL knees, and reconstructed knees were found during passive conditions. Moreover, significant differences in translational tibiofemoral RoM during gait were found between contralateral knees and PCL reconstructed knees. Orange-colored lines highlight the knees with additional PLC reconstruction.

A significant increase in the range of passive rotational laxity at 90° knee flexion can be observed in the PCL insufficient knees compared to the contralateral knees (*p* = 0.03). Similarly, a significant reduction in passive rotational laxity was observed in the PCL reconstructed knees when compared to the preoperative PCL insufficient state (*p* = 0.02). No significant differences were observed between PCL reconstructed and contralateral knees (Figure 3A).

### Passive translational knee joint laxity

Passive posterior translation was significantly higher in PCL insufficient knees compared to the contralateral knees (*p* < 0.01). A significant reduction in passive translational laxity was observed between PCL reconstructed knees and PCL insufficient knees (*p* < 0.01). The values after PCL reconstruction were however still significantly higher (*p* < 0.01) when compared to the contralateral knees (Figure 3C).

### Tibiofemoral rotational motion during level walking

**FIGURE 4:**
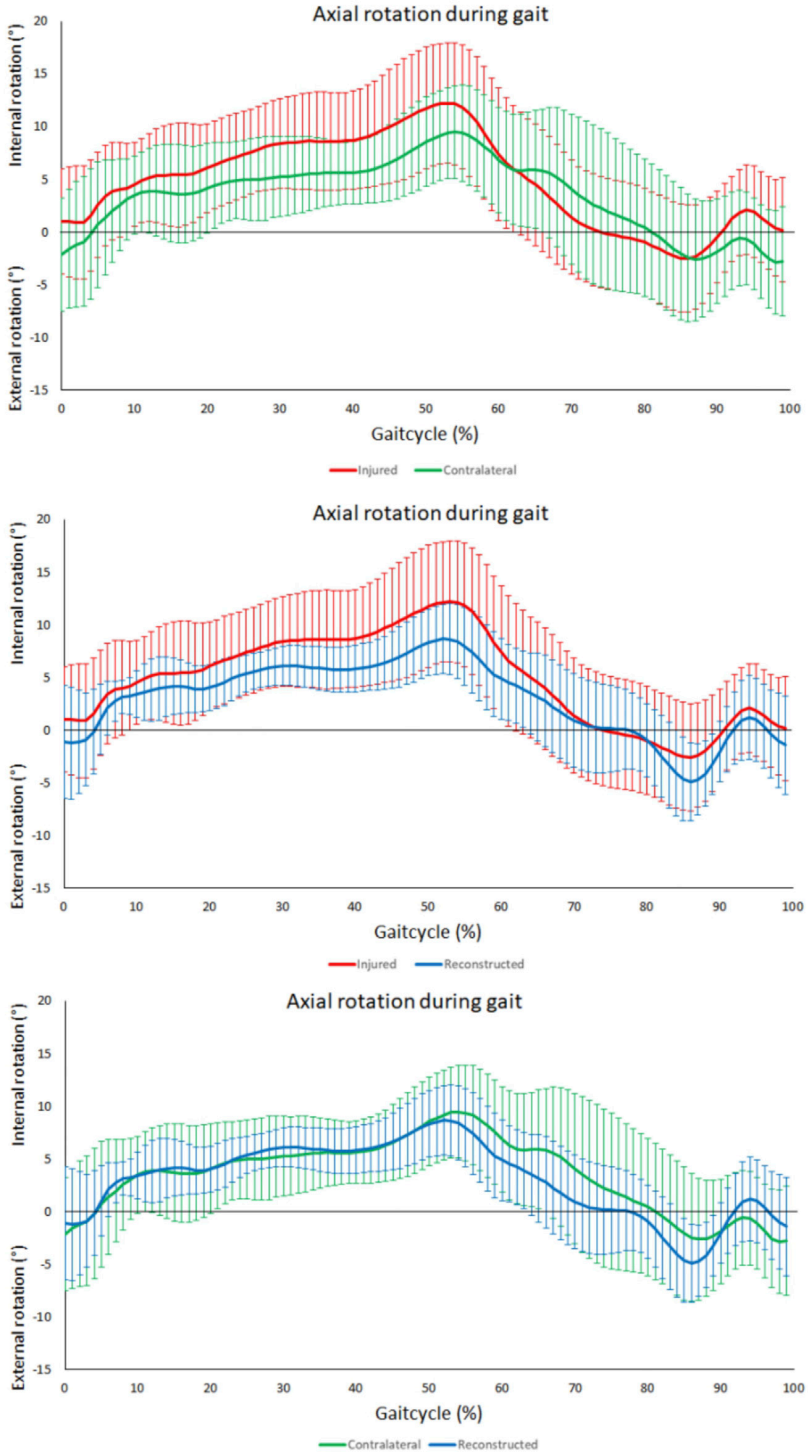
Tibiofemoral axial rotation during gait. From top to bottom; comparison between contralateral and PCL insufficient knees, comparison between PCL insufficient and reconstructed knees and comparison between contralateral and reconstructed knees. No significant differences throughout the gait cycle could be observed between the PCL insufficient, contralateral and reconstructed knees. Increased internal rotation in the PCL insufficient knees could be observed during the terminal stance (20%–50%) of the gait cycle.

The range of rotational motion during level walking showed no significant differences between PCL insufficient knees and contralateral knees (*p* = 0.31), PCL insufficient and reconstructed knees (*p* = 0.29), and reconstructed and contralateral knees (*p* = 0.47) (Figure 3B). Tibial positioning in PCL insufficient knees tended to be more internally rotated position than both to the contralateral side and PCL reconstructed knees (20%–50% of the gait cycle), but there were no significant differences along the complete gait cycle (Figure 4).

### Tibiofemoral anterior-posterior motion during level walking

**FIGURE 5:**
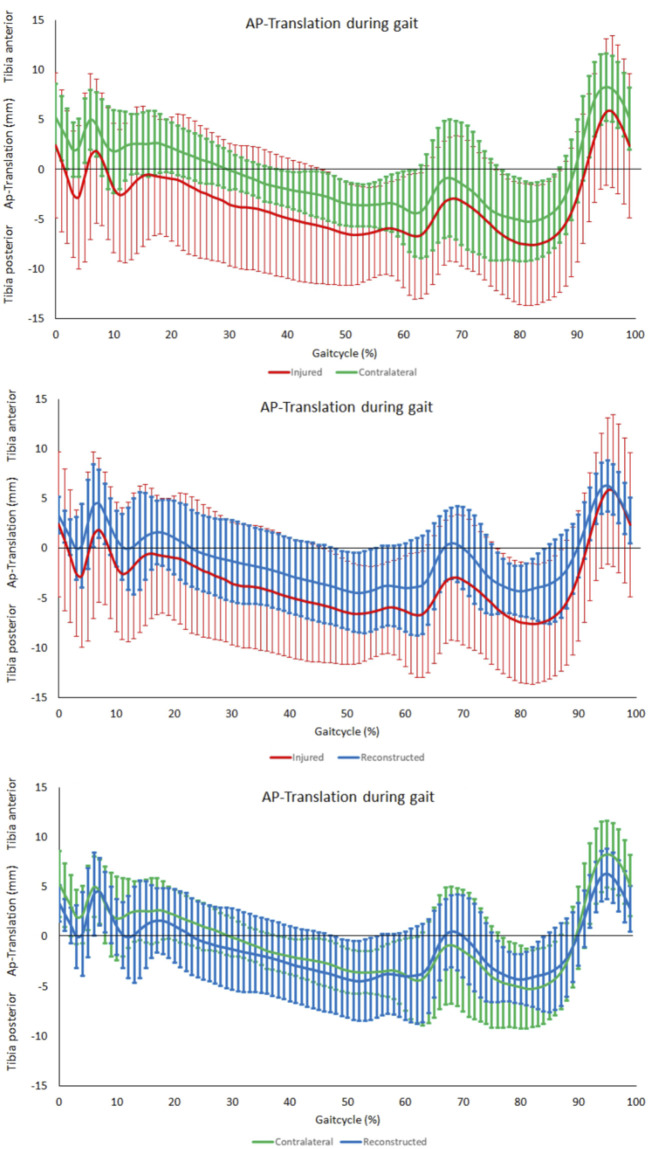
AP-Translation during gait. From top to bottom; comparison between contralateral and PCL insufficient knees, comparison between PCL insufficient knees and reconstructed knees and comparison between contralateral and reconstructed knees.

No significant differences were observed in the assessment of the range anterior-posterior tibiofemoral motion during level walking between the PCL insufficient and contralateral knees (*p* = 0.44). There was however a significant reduction after PCL reconstruction when compared to the contralateral knees (*p* < 0.01) but no significance when compared to the PCL insufficient knees (*p* = 0.07) (Figure 4D). The tibia was in a more posterior position in the PCL insufficient knees compared to the contralateral and PCL reconstructed knees, but there were no significant differences along the complete gait cycle (Figure 5).

### Knee joint extension-flexion angle analysis during level walking

**FIGURE 6:**
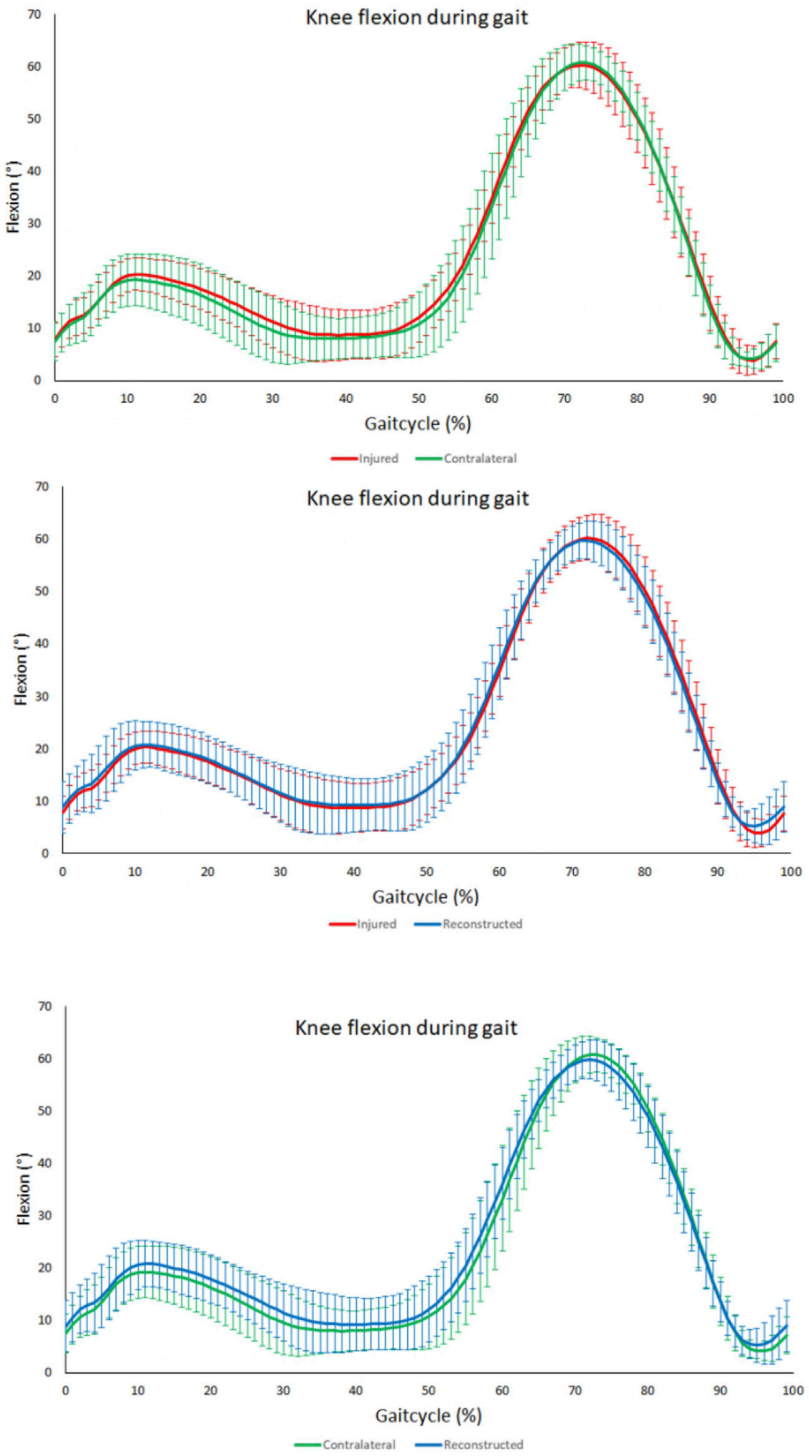
Knee flexion angle during gait. From top to bottom; comparison between contralateral and PCL insufficient knees, comparison between PCL insuffcient and reconstructed knees and comparison between contralateral and reconstructed knees. No significant differences throughout the gait cycle could be observed between the PCL insufficient, contralateral and reconstructed knees.

No significant differences were noted for the range of flexion over the entire gait cycle, the maximum flexion angle, the maximum extension angle, the maximum extension angle at mid stance (10%–30% of the gait cycle) and the maximal flexion at initial swing phase (60%–75%) (Figure 6).

## Discussion

This study provides for the first time an extensive prospective biomechanical *in vivo* analysis of patients undergoing PCL single-bundle reconstruction. The most important finding of this study was, that PCL reconstructed knees showed a significantly reduced range of anterior-posterior tibiofemoral motion over the entire gait cycle when compared to the contralateral knees. Hence, the hypothesis, that PCL reconstructed knees do not show significantly altered knee kinematics during level walking in comparison to contralateral knees, can be refuted for our patient cohort. Furthermore, a significant reduction of increased passive translational tibiofemoral laxity and a significant reduction of increased passive rotational tibiofemoral laxity was observed 6 months after PCL single-bundle reconstruction. Nevertheless, because of a residual higher passive tibiofemoral AP-laxity of the PCL reconstructed knees in comparison to the contralateral knees, the hypothesis, that PCL single-bundle reconstruction with optional PLC reconstruction restores increased passive tibiofemoral AP laxity and increased passive tibiofemoral rotational laxity in patients with PCL insufficiency, could not be fully confirmed in our patient cohort. No significant differences in the range of rotational tibiofemoral motion or in the range of knee flexion over the entire gait cycle could be shown.

Increased mean side-to-side difference of passive tibiofemoral AP-laxity measured via stress radiography was reduced from 12.1 mm (±4.4 mm) preoperatively to 4.3 mm (±1.8 mm) postoperatively. These results are in line with those previously reported in the literature following sinlge bundle PCL reconstruction, although passive tibiofemoral AP-laxity could not be fully restored (Lien et al., 2010; Zhang et al., 2010; Gwinner et al., 2019; Yoon et al., 2019). Some studies reported a higher reduction of passive tibiofemoral translational laxity after PCL reconstruction with most of them using PCL double-bundle reconstruction (Kim et al., 2009; Yoon et al., 2011; Jain et al., 2016). LaPrade et al. achieved an average reduction from 11.6 mm (±3.5 mm) preoperatively to 1.6 mm (±2.0 mm) postoperatively at a mean of 3 years after PCL double-bundle reconstruction (LaPrade et al., 2018). Concordant with these results, previous biomechanical studies have demonstrated a greater reduction of passive translational tibiofemoral laxity after PCL double-bundle reconstruction compared to PCL single-bundle reconstruction (Wijdicks et al., 2013; Chahla et al., 2017; Lee et al., 2017). Two recent meta-analyses from Krott et al. and Migliorini et al. have been able to confirm these findings in clinical studies (Krott et al., 2022; Migliorini et al., 2022). Nevertheless, the current consensus is that the superiority of the PCL double-bundle reconstruction technique over the PCL single-bundle reconstruction technique in terms of clinical outcome has not yet been demonstrated (Tucker et al., 2018; Krott et al., 2022; Migliorini et al., 2022).

Quantifying passive tibiofemoral translational laxity via stress radiography is an established procedure and the only objective measurement to evaluate the biomechanical success of PCL reconstruction in clinical practice (Jung et al., 2006a). In our study, patients undergoing PCL single bundle reconstruction had, in addition to significantly increased passive tibiofemoral translational laxity, a significantly increased passive tibiofemoral rotational laxity. This result reflects the current knowledge of PCL insufficiency and the relationship with passive tibiofemoral rotational laxity. The posterior cruciate ligament is the secondary stabilizer of tibiofemoral external rotation (Sekiya et al., 2005). Increased passive tibiofemoral internal rotation laxity in PCL insufficient knees, especially at more than 90° knee flexion was also demonstrated in a cadaveric study by Wijdicks et al. (Wijdicks et al., 2013). The present study demonstrates a reduction of the increased passive tibiofemoral rotational laxity to the level of the contralateral knees after PCL single-bundle reconstruction at 90° of knee flexion. The patients who received an additional PLC reconstruction certainly contributed to this result. It has already been demonstrated in biomechanical cadaveric studies that the combination of PCL and PLC reconstruction can restore increased passive tibiofemoral rotational laxity. Krudwig et al. demonstrated in their biomechanical study on ten cadaveric knees after combined transection of the PCL and the PLC a restoration of increased passive tibiofemoral laxity using PCL single-bundle reconstruction in combination with PLC reconstruction in modified Larson technique (Krudwig et al., 2002). Apsingi et al. also observed a complete restoration of passive tibiofemoral external rotation laxity to the level of the intact state using PCL single-bundle reconstruction in combination with PLC reconstruction in modified Larson technique in their cadaveric study (Apsingi et al., 2008b). The *in vivo* data of our study confirm the results of these biomechanical studies. Zorzi et al. (Zorzi et al., 2013) recorded in their clinical study a restoration of increased tibiofemoral external rotation laxity to the intact state in 17 of 19 patients, who had also received a combined PCL single-bundle reconstruction with a PLC reconstruction using the modified Larson technique. This study was performed with the dial test and is in accordance with our findings. In a cadaveric study by Drenck et al., increased passive tibiofemoral external rotation laxity could not be restored by PCL double-bundle reconstruction in combination with additional PLC reconstruction using a modified Larson technique (Drenck et al., 2021). A possible explanation for this may be the *in vivo* nature of our study, or the differences in applied torque in both studies. A recent meta-analysis of clinical trials by Boksh et al. rated fibular-based techniques like the modified Larson technique in combination with PCL reconstruction as an effective method to restore increased tibiofemoral rotational laxity for PLC and PCL knee injuries, which reflects our results (Boksh et al., 2023). Nevertheless, anatomic posterolateral corner reconstruction is the preferred technique for posterolateral corner reconstruction in a current expert consensus statement (Chahla et al., 2019).

The analysis of knee kinematics showed no significant differences in the range of anterior-posterior tibiofemoral motion during level walking between the PCL insufficient knees and contralateral knees. This is consistent with previous study results. Goyal et al. (Goyal et al., 2012) found no differences between the involved and uninvolved legs of three PCL-deficient patients regarding the tibiofemoral translation during level walking measured via Dynamic Stereo X-Ray. However, they found a higher dorsal subluxation of the injured knee compared to the contralateral knee during stair ascent and in the swing phase during running. Corresponding to our data, Orita et al. (Orita et al., 2015) recognized even a reduction of tibiofemoral AP translation by comparing PCL-deficient knees with a healthy control group during level walking. They could observe a reduction of approximately 7 mm in tibiofemoral AP translation at 39%–52% of the gait cycle, which was not observed in our study. This could probably be due to the fact that they compared the PCL-deficient knees with a healthy control group and not with the contralateral limb as we did in the present study. Nevertheless, we could detect a significant reduction in the range of anterior-posterior tibiofemoral motion after PCL reconstruction when compared to the contralateral knees.

To date, very few studies have analyzed gait biomechanics of patients after PCL reconstruction, with none of them analyzing the range of anterior-posterior tibiofemoral motion (Tibone et al., 1988; Hart et al., 2009; Brisson et al., 2021). Brisson et al. (Brisson et al., 2021) found no difference between reconstructed and contralateral limbs for any parameter in their analysis of knee joint kinematics during level walking 8 years after PCL reconstruction. We analyzed gait kinematics 6 months after posterior cruciate ligament single bundle reconstruction to get insights during the rehabilitation phase. Similar to their findings, we obtained no significant differences in the range of knee flexion and in the range of tibiofemoral rotation over the entire gait cycle. Contrary to our findings Hart et al. (Hart et al., 2009) observed a 33% reduced range of motion in knee flexion during level walking in their cohort by comparing reconstructed knees with the contralateral knees. This discrepancy can likely be explained by the different patient cohorts. Hart et al. (Hart et al., 2009) analyzed a patient cohort with reconstructed multiple ligament injuries. Of the 25 patients analyzed, no patient had an isolated PCL injury and only four patients had a combined PCL and PLC injury. Similar findings to ours have also been reported for patients undergoing anterior cruciate ligament (ACL) reconstruction in the setting of anterior cruciate ligament deficiency. Boeth et al. (Boeth et al., 2013) detected a significantly lower range of tibiofemoral anterior-posterior translation over the entire gait cycle of ACL-ruptured knees compared to contralateral joints. Shabani et al. (Shabani et al., 2015) could observe no significant difference in anterior-posterior femorotibial translation when comparing ACL reconstructed knees with ACL deficient knees, contralateral knees, and a healthy control group. A possible explanation could be adaptive strategies with gait adaptations through active muscle co-contraction. A previous study by Cain et al. (Cain and Schwab, 1981) has shown that early contraction of the quadriceps muscle during gait is used in a PCL-deficient subject as an adaptive muscular compensation, which allegedly reduces the load on the PCL. Early activation of the gastrocnemius-soleus complex has also been demonstrated in patients with PCL insufficiency, reinforcing the assumption of compensatory muscle activation (Tibone et al., 1988; Inoue et al., 1998). In patients with ACL injuries and increased anterior tibial translation, a “quadriceps avoidance pattern” was observed during gait (Papadonikolakis et al., 2003). Thus, the anterior tibial translation and consequently the load on the ACL could be reduced. So, it is also possible that muscular compensatory mechanisms are active in patients after PCL single-bundle reconstruction leading to a decreased range of anterior-posterior tibiofemoral motion during level walking. Altered muscle forces can lead to increased joint loading and therefore can lead to degenerative changes in the joint (Andriacchi et al., 2004). Previous studies have already shown that certain gait biomechanics are associated with increased odds of osteoarthritis onset (D Souza et al., 2021). Therefore, it is crucial to gain a more precise understanding of altered gait biomechanics after PCL reconstruction and their impact on the development of posttraumatic osteoarthritis.

We acknowledge several limitations of this study. First to mention is the inclusion of patients with an additional PLC reconstruction. We deliberately included these patients because most patients suffering a PCL injury have a concomitant PLC injury (Anderson et al., 2018). Another limitation is the absence of a healthy control group, which is due to ethical concerns regarding the application of X-ray radiation during fluoroscopic measurements. In the context of the measurement methods, it is important to mention, that the accuracy of the marker-based technique used for gait analysis is reduced compared to the fluoroscopy technique used for the passive tibiofemoral rotational measurements and stress radiography used for the passive translational tibiofemoral measurements. However, through the application of the OCST approach during marker-based measurements a reduction of around 50% on skin marker artifacts can be achieved.

The early time point of postoperative measurement has to be mentioned as well. However, the timing was intentionally chosen to analyze gait patterns during the late phase of rehabilitation, when the patients already are moving with full weight-bearing during their daily activities and therefore delivering insights as a basis for adjusting rehabilitation protocols. Nevertheless, the readers have to take into account, that passive tibiofemoral translational laxity increases over time after PCL single bundle reconstruction (Gwinner et al., 2019). Another limitation is the small number of analyzed patients, which also limits the significance of the statistical analysis. This can be justified by our inclusion criteria and the fact that the incidence of PCL injuries is relatively small and has decreased over the last years (Orchard and Seward, 2009). Moreover, an acute isolated PCL injury can be treated in most cases conservatively (Kew et al., 2022) or if surgical treatment is indicated with ligament repair through suture tape augmentation without the need for PCL reconstruction (Hopper et al., 2019).

## Conclusion

Patients undergoing PCL single-bundle reconstruction showed postoperatively altered knee kinematics during level walking. The RoM of tibiofemoral anterior-posterior translation during level walking was significantly reduced compared to the contralateral knees. The reason could be postoperative gait adaptations through muscle co-contraction, which could be a possible cause for joint degeneration after PCL single-bundle reconstruction. Increased passive tibiofemoral translational laxity could be significantly reduced, but not fully restored to the intact state. Increased passive tibiofemoral rotational laxity at 90° knee flexion could be restored through PCL single-bundle reconstruction with optional PLC reconstruction. This indicates that PCL single-bundle reconstruction with optional PLC reconstruction is a reasonable treatment option for PCL insufficient knees with a remaining lack of full restoration of biomechanics in the active and passive state.

## Data Availability

The raw data supporting the conclusion of this article will be made available by the authors, without undue reservation.

## Abbreviations

(PCL), Posterior cruciate ligament; (PCL-SR), Posterior cruciate ligament single-bundle reconstruction; (PLC), Posterolateral corner; (AP), Anterior-posterior; (CL), Contralateral; (KOOS), Knee Injury and Osteoarthritis Outcome Score; (SF-36), Short Form 36; (Pseudo-CT), Pseudo compute tomography; (MRI), Magnetic resonance imaging; (TR), Time to repeat; (TE), Time to echo; (SSM), Statistical shape models; (SSIM), Shape and intensity model; (3D/2D), Three dimensional/two dimensional; (OCST), Optimal common shape technique; (SARA), Symmetrical axis of rotation approach; (SCoRE), Symmetrical center of rotation estimation; (RoM), Range of motion; (ACL), Anterior cruciate ligament; We wish to thank all the subjects who took part in this study.
